# Retraction: Upregulation of microRNA-148a inhibits proliferation, invasion and migration while promoting apoptosis of cervical cancer cells by downregulating RRS1

**DOI:** 10.1042/BSR-20181815_RET

**Published:** 2020-07-22

**Keywords:** Cervical cancer, microRNA-148a, RRS1, Proliferation, Invasion, Migration

This Retraction follows an Expression of Concern relating to this article previously published by Portland Press.

This article is being retracted from Bioscience Reports at the request of the Editor-in-Chief and the Editorial Board following receipt of a notification from a reader, alerting the Editorial Board to multiple manipulations in Figures 6A and 6C, as well as duplications in Figure 10A and 10C.

The authors have been contacted in regard to the retraction, and have not responded to the Journal's queries and the concerns raised. Given the extent of the issues raised, Editorial Board stand by the decision to retract the article.

